# Crystal structure of 2-[chloro­(4-meth­oxy­phen­yl)meth­yl]-2-(4-meth­oxy­phen­yl)-5,5-di­methyl­cyclo­hexane-1,3-dione

**DOI:** 10.1107/S2056989016002085

**Published:** 2016-02-06

**Authors:** Saloua Chelli, Konstantin Troshin, Sami Lakhdar, Herbert Mayr, Peter Mayer

**Affiliations:** a Department Chemie und Biochemie, Ludwig-Maximilians-Universität, Butenandtstrasse 513, D-81377 München, Germany

**Keywords:** crystal structure, weak C—H⋯O and C—H⋯Cl inter­actions

## Abstract

In the title compound, C_23_H_25_ClO_4_, the cyclo­hexane ring adopts a chair conformation with the 4-meth­oxy­phenyl substituent in an axial position and the chloro­(4-meth­oxy­phen­yl)methyl substituent in an equatorial position. The packing features inversion dimers formed by pairs of C—H⋯O contacts and strands along [100] and [010] established by further C—H⋯O and C—H⋯Cl contacts, respectively.

## Abstract 2

One of the methyl groups and the 4-meth­oxy­phenyl substituent are in axial positions and the chloro­(4-meth­oxy­phen­yl)methyl substituent is in the equatorial position of the cyclo­hexane ring which adopts a chair conformation. The packing features inversion-symmetric dimeric units and strands along [100] and [010] established by weak C—H⋯O and C—H⋯Cl contacts.

## Chemical context   

Iodo­nium ylides, a subclass of hypervalent iodine compounds (Zhdankin & Stang, 2008 ▸), have a variety of synthetic applications due to their versatile reactivity pattern. The known transformations of these reagents include decomposition (Moriarty *et al.*, 2008 ▸; Lee & Jung, 2002 ▸) in various solvents, transylidation reactions (Hadjiarapoglou & Varvoglis, 1988 ▸), C–H insertion reactions (Adam *et al.*, 2003 ▸; Batsila *et al.*, 2003 ▸) and intra- and inter­molecular cyclo­addition reactions under photochemical, thermal, or metal-catalysed activation (Goudreau *et al.*, 2009 ▸). During our studies on the reactions of iodo­nium ylides with stabilized carbenium ions, we obtained the title compound, the structure of which provides valuable information on the mechanism of these reactions that will be discussed in a separate paper.
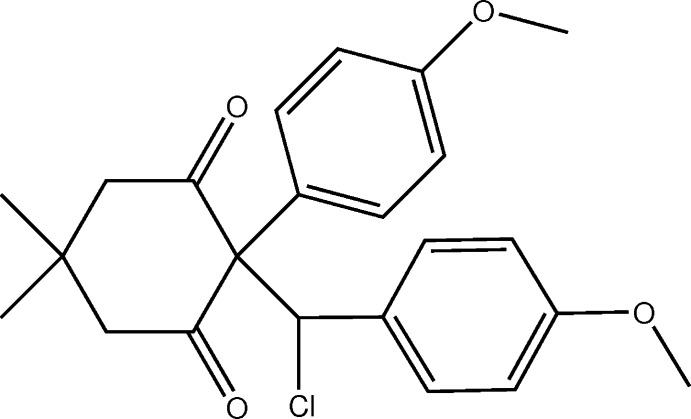


## Structural commentary   

**Figure 1:**
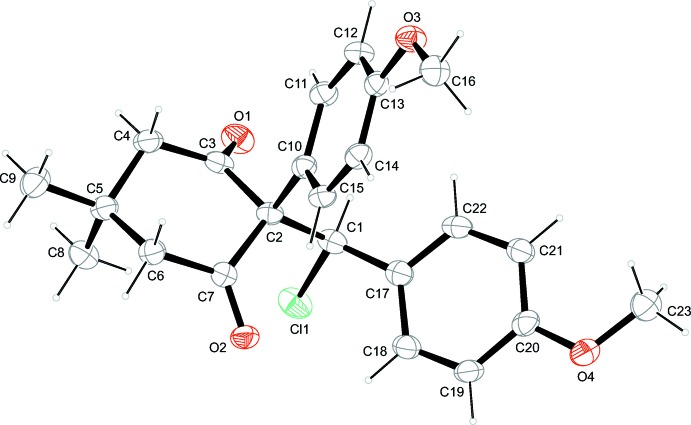
The mol­ecular structure of the title compound. Displacement ellipsoids are drawn at the 50% probability level.

The title compound (Fig. 1 ▸) comprises three six-membered rings: two benzene rings and a cyclo­hexane ring adopting a chair-conformation, with puckering amplitude *Q* = 0.5247 (19) Å and *θ* = 167.6 (2)° (Boeyens, 1978 ▸; Cremer & Pople, 1975 ▸). The maximum deviation from the mean plane is 0.269 (2) Å for atom C5. The 4-meth­oxy­phenyl substituent is in an axial position, while the chloro­(4-meth­oxy­phen­yl)methyl substituent is in an equatorial position. As expected, the two keto-C atoms are substituted in a trigonal–planar fashion. The C1—Cl1 bond is almost parallel to the axial C5—C8 bond (methyl substituent) with a C8—C5—C1—Cl1 torsion angle of −5.88 (11)°. The methyl C16 and the meth­oxy C23 carbon atoms have maximum deviations from the respective benzene rings, C10–C16 and C17–C22, of 0.085 (2) and 0.057 (2) Å, respectively, and hence are almost coplanar with them. The two benzene rings are inclined to one another by 41.38 (6)° and to the mean plane of the cyclohexane ring by 75.27 (9) and 43.40 (8)°, respectively.

## Supra­molecular features   

**Figure 2:**
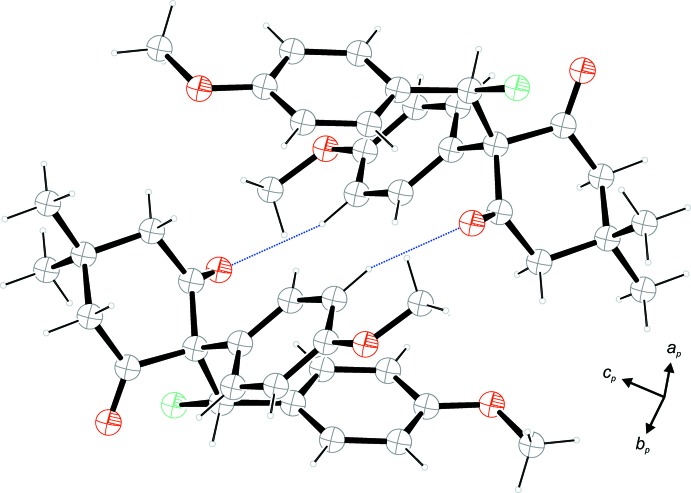
A view of the inversion dimer formed by a pair of weak C—H⋯O contacts (blue dotted lines).

**Figure 3:**
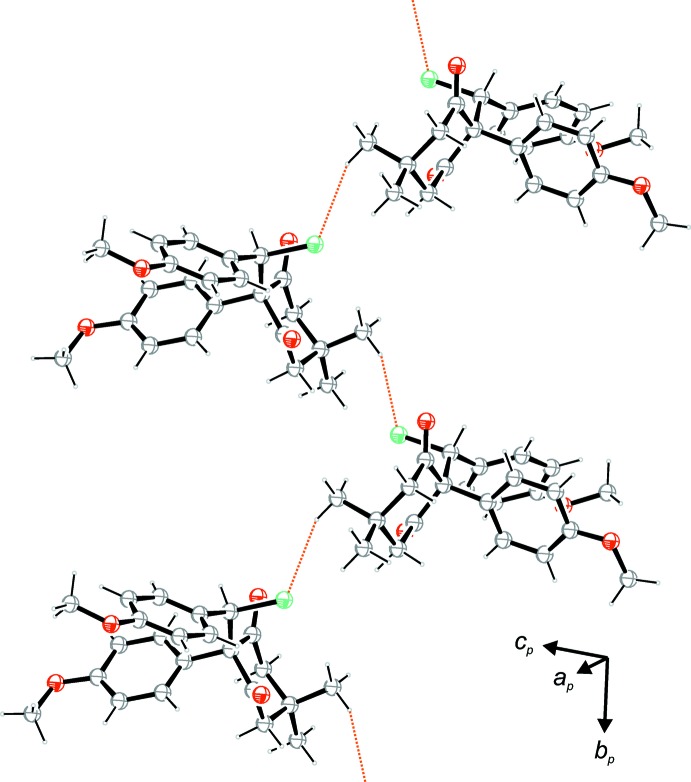
A view of the strands along [010] formed by weak C—H⋯Cl contacts (orange dotted lines).

**Figure 4:**
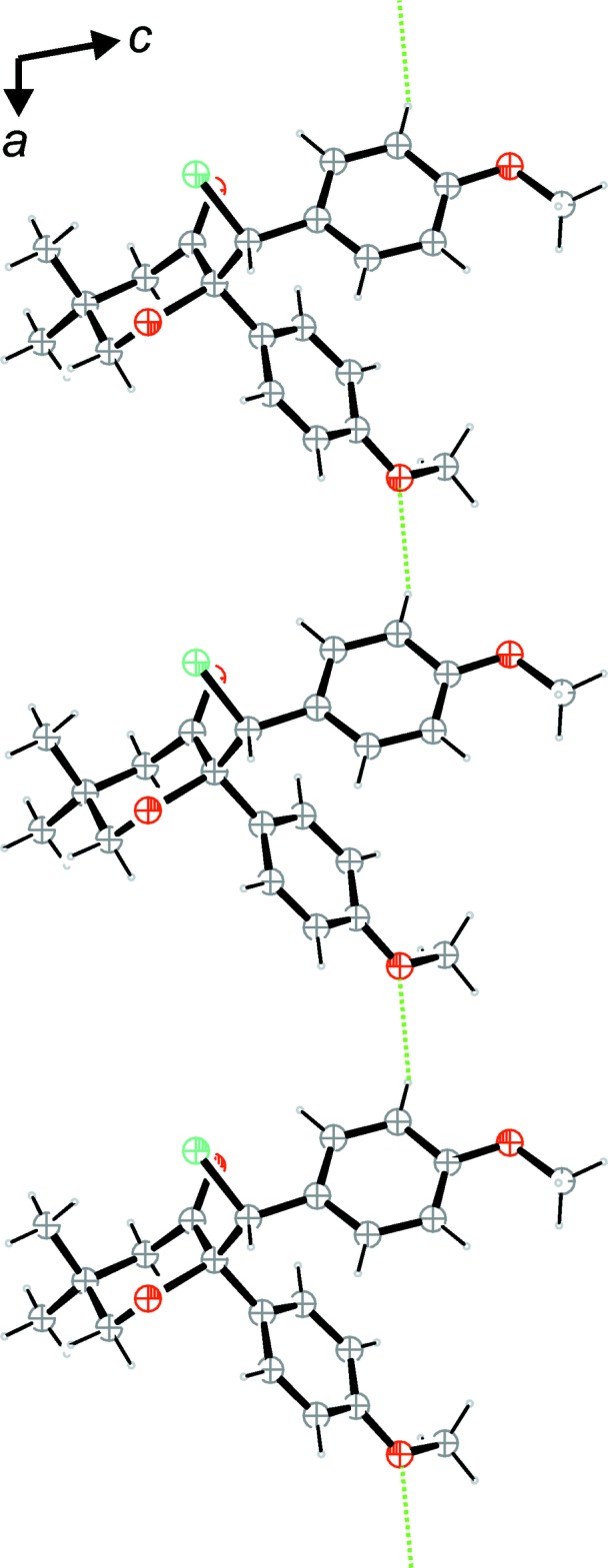
A view along [010] of the strands along [100] formed by weak C—H⋯O contacts (green dotted lines).

**Figure 5:**
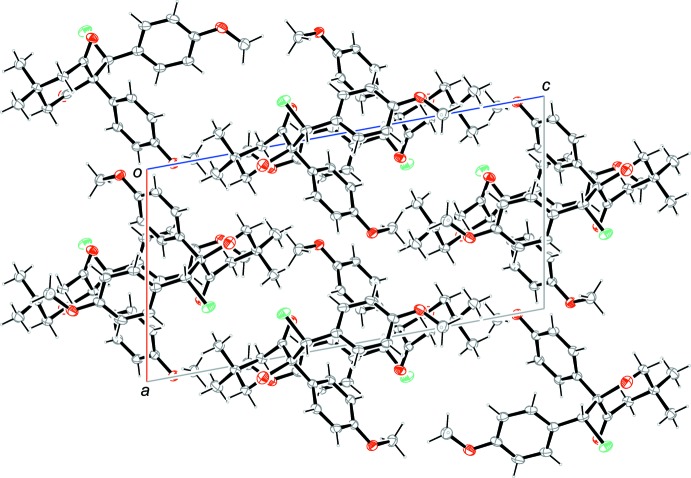
Packing diagram of the title compound viewed along [010]. For clarity, all the weak inter­actions have been omitted.

The packing of the title compound manifests weak C—H⋯O and C—H⋯Cl contacts (Table 1 ▸), while π-stacking and C—H⋯π inter­actions are not present. Pairs of contacts of the type C14—H14⋯O2 between the benzene ring and a keto-group lead to the formation of inversion dimers with an 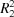
(14) ring motif (Fig. 2 ▸). Strands along [010] are established by weak C8–H8*C*⋯Cl1 contacts between the axial-oriented methyl substituent of the cyclo­hexane ring and the chloro substituent (Fig. 3 ▸). Finally, strands along [100] are formed by C19—H19⋯O3 contacts between the benzene ring (C17–C22) and the methoxy group on benzene ring C10–C16 (Fig. 4 ▸). The full packing including cell outlines is shown in Fig. 5 ▸.

## Database survey   

A CSD database (Version 5.36; Groom & Allen, 2014 ▸) search has been conducted for the three structure fragments *A*, *B* and *C* depicted in the following scheme.
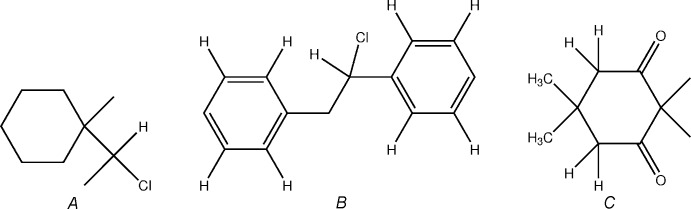


The search for fragment *A* yielded 21 hits; however, in 20 of them the cyclo­hexane ring is part of an annulated ring system and in the remaining hit it is part of a spiro-compound. Since none of the hits is really closely related to the title compound, they are not cited in detail. The search for fragment *B* led to six hits with the CSD refcodes CBZPOX (Noordik & Cillissen, 1981 ▸), IYISAL (Sparr & Gilmour, 2011 ▸), PAQKAV (Nair *et al.*, 2012 ▸), POMZOH (Unruh *et al.*, 2008 ▸), UREKEI (Betz *et al.*, 2011 ▸) and YUZPOZ (Kalyani *et al.*, 2010 ▸). Finally, the search for fragment *C* comprising the 5,5-di­methyl­cyclo­hexane-1,3-dione moiety produced 25 hits. In merely two of them fragment *C* is part of a non-spiro compound comparable to the title compound: CSD refcodes CETMCD (Roques *et al.*, 1976 ▸) and FAWDEM (Ochiai *et al.*, 1986 ▸).

## Synthesis and crystallization   

Zinc chloride (114.2 mg, 699 µmol), tetra­butyl­ammonium chloride (190.2 mg, 684 µmol), diethyl ether (0.10 ml) and phenyl­iodo­nium-4,4-di­methyl­cyclo­hexane-2,6-dione (568.6 mg, 1.66 mmol) were dissolved in di­chloro­methane (6 ml) and cooled to 195 K. Then 4,4′-di­meth­oxy­benzhydryl chloride (417.2 mg, 1.59 mmol) in di­chloro­methane (4 ml) was added dropwise. The reaction solution was stirred at 195 K for 2 h. The resulting mixture was quenched with 2 *M* aqueous ammonia. Diethyl ether was added to the organic phase followed by washing with water and brine, drying (MgSO_4_), and evaporation of the solvents in a vacuum. The crude product was recrystallized from diethyl ether/pentane (1:1 *v*/*v*) affording the title compound (394 mg, 982 µmol; yield 62%).

## Refinement   

Crystal data, data collection and structure refinement details are summarized in Table 2 ▸. All H atoms were positioned geometrically (C—H = 0.98 Å for methyl-H, 0.99 Å for C—H_2_, 1.00 Å for aliphatic C—H, 0.95 Å for aromatic H) and treated as riding on their parent atoms, with *U*_iso_(H) = 1.2*U*_eq_(C) or 1.*5*U_eq_(C) for methyl H atoms. The methyl groups were allowed to rotate along the C—C bonds to best fit the experimental electron density.

## Supplementary Material

Crystal structure: contains datablock(s) I, global. DOI: 10.1107/S2056989016002085/rz5184sup1.cif

Structure factors: contains datablock(s) I. DOI: 10.1107/S2056989016002085/rz5184Isup2.hkl

Supporting information file. DOI: 10.1107/S2056989016002085/rz5184Isup3.cml

CCDC reference: 1451618

Additional supporting information:  crystallographic information; 3D view; checkCIF report

## Figures and Tables

**Table 1:** Hydrogen-bond geometry (Å, °)

*D*—H⋯*A*	*D*—H	H⋯*A*	*D*⋯*A*	*D*—H⋯*A*
C8—H8*C*⋯Cl1^i^	0.98	2.81	3.745 (2)	159
C14—H14⋯O2^ii^	0.95	2.52	3.394 (2)	153
C19—H19⋯O3^iii^	0.95	2.56	3.470 (2)	161

Symmetry codes: (i) 
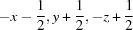
; (ii) 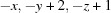; (iii) 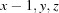.

**Table 2:** Experimental details

Crystal data	
Chemical formula	C_23_H_25_ClO_4_
*M*_r_	400.88
Crystal system, space group	Monoclinic, *P*2_1_/*n*
Temperature (K)	100
*a*, *b*, *c* (Å)	10.0235 (5), 11.1997 (6), 19.0655 (12)
β (°)	100.429 (6)
*V* (Å^3^)	2104.9 (2)
*Z*	4
Radiation type	Mo *K*α
μ (mm^−1^)	0.21
Crystal size (mm)	0.40 × 0.32 × 0.22

Data collection	
Diffractometer	Oxford Diffraction XCalibur3
Absorption correction	Multi-scan (*CrysAlis PRO*; Agilent, 2014 ▸)
*T*_min_, *T*_max_	0.982, 1.000
No. of measured, independent and observed [*I* > 2σ(*I*)] reflections	11293, 4283, 3355
*R*_int_	0.031
(sin θ/λ)_max_ (Å^−1^)	0.625

Refinement	
*R*[*F* ^2^ > 2σ(*F* ^2^)], *wR*(*F* ^2^), *S*	0.040, 0.102, 1.03
No. of reflections	4283
No. of parameters	257
H-atom treatment	H-atom parameters constrained
Δρ_max_, Δρ_min_ (e Å^−3^)	0.27, −0.30

Computer programs: *CrysAlis PRO* (Agilent, 2014 ▸), *SIR97* (Altomare *et al.*, 1999 ▸), *SHELXL2014* (Sheldrick, 2015 ▸), *ORTEPIII* (Burnett & Johnson, 1996 ▸) and *PLATON* (Spek, 2009 ▸).

## supplementary crystallographic information

### Crystal data

**Table:**

C_23_H_25_ClO_4_	*F*(000) = 848
*M**_r_* = 400.88	*D*_x_ = 1.265 Mg m^−^^3^
Monoclinic, *P*2_1_/*n*	Mo *K*α radiation, λ = 0.71069 Å
*a* = 10.0235 (5) Å	Cell parameters from 3451 reflections
*b* = 11.1997 (6) Å	θ = 4.4–28.5°
*c* = 19.0655 (12) Å	µ = 0.21 mm^−^^1^
β = 100.429 (6)°	*T* = 100 K
*V* = 2104.9 (2) Å^3^	Block, colourless
*Z* = 4	0.40 × 0.32 × 0.22 mm

### Data collection

**Table:**

Oxford Diffraction XCalibur3 diffractometer	4283 independent reflections
Radiation source: fine-focus sealed tube	3355 reflections with *I* > 2σ(*I*)
Detector resolution: 15.9809 pixels mm^-1^	*R*_int_ = 0.031
ω scans	θ_max_ = 26.4°, θ_min_ = 4.2°
Absorption correction: multi-scan (*CrysAlis PRO*; Agilent, 2014)	*h* = −12→9
*T*_min_ = 0.982, *T*_max_ = 1.000	*k* = −13→13
11293 measured reflections	*l* = −23→22

### Refinement

**Table:**

Refinement on *F*^2^	0 restraints
Least-squares matrix: full	Hydrogen site location: inferred from neighbouring sites
*R*[*F*^2^ > 2σ(*F*^2^)] = 0.040	H-atom parameters constrained
*wR*(*F*^2^) = 0.102	*w* = 1/[σ^2^(*F*_o_^2^) + (0.0382*P*)^2^ + 0.9962*P*] where *P* = (*F*_o_^2^ + 2*F*_c_^2^)/3
*S* = 1.02	(Δ/σ)_max_ < 0.001
4283 reflections	Δρ_max_ = 0.27 e Å^−^^3^
257 parameters	Δρ_min_ = −0.30 e Å^−^^3^

### Special details

**Table:**

Experimental. Absorption correction: *CrysAlis PRO* (Agilent, 2014), Empirical absorption correction using spherical harmonics, implemented in SCALE3 ABSPACK scaling algorithm.
Geometry. All e.s.d.'s (except the e.s.d. in the dihedral angle between two l.s. planes) are estimated using the full covariance matrix. The cell e.s.d.'s are taken into account individually in the estimation of e.s.d.'s in distances, angles and torsion angles; correlations between e.s.d.'s in cell parameters are only used when they are defined by crystal symmetry. An approximate (isotropic) treatment of cell e.s.d.'s is used for estimating e.s.d.'s involving l.s. planes.

### Fractional atomic coordinates and isotropic or equivalent isotropic displacement parameters (Å^2^)

**Table:**

	*x*	*y*	*z*	*U*_iso_*/*U*_eq_	
Cl1	−0.20291 (4)	0.62036 (4)	0.34454 (3)	0.03325 (14)	
O1	0.08165 (13)	0.59745 (11)	0.29198 (7)	0.0321 (3)	
C1	−0.04506 (17)	0.66193 (15)	0.40194 (9)	0.0251 (4)	
H1	0.0144	0.5898	0.4047	0.030*	
O2	−0.16483 (12)	0.88985 (11)	0.36243 (6)	0.0272 (3)	
C2	0.02730 (16)	0.75977 (14)	0.36481 (9)	0.0216 (4)	
O3	0.49570 (12)	0.88680 (11)	0.56782 (6)	0.0280 (3)	
C3	0.07764 (16)	0.70477 (16)	0.29949 (9)	0.0245 (4)	
O4	−0.10111 (12)	0.75401 (12)	0.68822 (7)	0.0326 (3)	
C4	0.12691 (17)	0.79029 (17)	0.24936 (10)	0.0281 (4)	
H4A	0.1472	0.7457	0.2077	0.034*	
H4B	0.2121	0.8279	0.2738	0.034*	
C5	0.02225 (17)	0.88830 (15)	0.22329 (9)	0.0251 (4)	
C6	−0.01011 (17)	0.95395 (15)	0.28892 (9)	0.0248 (4)	
H6A	0.0727	0.9948	0.3137	0.030*	
H6B	−0.0797	1.0157	0.2731	0.030*	
C7	−0.06089 (16)	0.87099 (15)	0.34059 (9)	0.0218 (3)	
C8	−0.10614 (18)	0.83159 (17)	0.18100 (10)	0.0301 (4)	
H8A	−0.1437	0.7749	0.2115	0.045*	
H8B	−0.0839	0.7894	0.1396	0.045*	
H8C	−0.1732	0.8940	0.1648	0.045*	
C9	0.0821 (2)	0.97616 (18)	0.17590 (10)	0.0355 (5)	
H9A	0.0155	1.0387	0.1593	0.053*	
H9B	0.1050	0.9336	0.1348	0.053*	
H9C	0.1643	1.0125	0.2033	0.053*	
C10	0.15436 (16)	0.80039 (15)	0.41729 (9)	0.0215 (3)	
C11	0.27367 (17)	0.73249 (15)	0.42612 (9)	0.0244 (4)	
H11	0.2779	0.6643	0.3970	0.029*	
C12	0.38505 (17)	0.76335 (15)	0.47659 (9)	0.0257 (4)	
H12	0.4654	0.7167	0.4818	0.031*	
C13	0.37996 (16)	0.86264 (15)	0.51996 (9)	0.0225 (4)	
C14	0.26248 (17)	0.93041 (15)	0.51276 (9)	0.0237 (4)	
H14	0.2580	0.9978	0.5425	0.028*	
C15	0.15099 (17)	0.89837 (15)	0.46128 (9)	0.0235 (4)	
H15	0.0706	0.9449	0.4562	0.028*	
C16	0.48994 (18)	0.98232 (17)	0.61706 (10)	0.0308 (4)	
H16A	0.4706	1.0574	0.5908	0.046*	
H16B	0.5772	0.9887	0.6497	0.046*	
H16C	0.4180	0.9663	0.6444	0.046*	
C17	−0.06513 (16)	0.68527 (15)	0.47733 (9)	0.0246 (4)	
C18	−0.17687 (17)	0.74582 (16)	0.49528 (10)	0.0291 (4)	
H18	−0.2472	0.7729	0.4585	0.035*	
C19	−0.18604 (18)	0.76657 (17)	0.56548 (10)	0.0312 (4)	
H19	−0.2625	0.8076	0.5766	0.037*	
C20	−0.08395 (17)	0.72774 (15)	0.62008 (10)	0.0261 (4)	
C21	0.02565 (17)	0.66535 (15)	0.60393 (10)	0.0266 (4)	
H21	0.0946	0.6367	0.6409	0.032*	
C22	0.03362 (17)	0.64505 (15)	0.53272 (10)	0.0261 (4)	
H22	0.1091	0.6023	0.5218	0.031*	
C23	−0.00265 (19)	0.70699 (18)	0.74517 (10)	0.0340 (4)	
H23A	−0.0006	0.6198	0.7415	0.051*	
H23B	−0.0268	0.7296	0.7909	0.051*	
H23C	0.0870	0.7394	0.7422	0.051*	

### Atomic displacement parameters (Å^2^)

**Table:**

	*U*^11^	*U*^22^	*U*^33^	*U*^12^	*U*^13^	*U*^23^
Cl1	0.0259 (2)	0.0275 (2)	0.0429 (3)	−0.00490 (17)	−0.00321 (18)	0.0016 (2)
O1	0.0347 (7)	0.0273 (7)	0.0327 (7)	0.0070 (5)	0.0017 (5)	−0.0067 (6)
C1	0.0197 (8)	0.0210 (9)	0.0334 (10)	0.0007 (6)	0.0014 (7)	0.0008 (7)
O2	0.0245 (6)	0.0263 (7)	0.0330 (7)	0.0066 (5)	0.0108 (5)	0.0034 (5)
C2	0.0195 (8)	0.0191 (8)	0.0262 (9)	0.0021 (6)	0.0043 (7)	−0.0014 (7)
O3	0.0231 (6)	0.0311 (7)	0.0286 (7)	0.0003 (5)	0.0014 (5)	−0.0054 (5)
C3	0.0177 (8)	0.0268 (9)	0.0273 (9)	0.0049 (7)	−0.0007 (7)	−0.0039 (8)
O4	0.0291 (7)	0.0366 (8)	0.0349 (7)	0.0034 (5)	0.0129 (5)	0.0039 (6)
C4	0.0215 (9)	0.0349 (10)	0.0286 (9)	0.0046 (7)	0.0068 (7)	−0.0031 (8)
C5	0.0231 (9)	0.0252 (9)	0.0279 (9)	0.0013 (7)	0.0074 (7)	−0.0001 (7)
C6	0.0248 (9)	0.0213 (9)	0.0291 (9)	0.0006 (7)	0.0066 (7)	0.0015 (7)
C7	0.0222 (8)	0.0207 (8)	0.0220 (8)	0.0000 (7)	0.0024 (6)	−0.0031 (7)
C8	0.0292 (10)	0.0283 (10)	0.0312 (10)	0.0038 (7)	0.0016 (7)	−0.0010 (8)
C9	0.0350 (11)	0.0382 (11)	0.0361 (11)	−0.0019 (8)	0.0138 (8)	0.0041 (9)
C10	0.0208 (8)	0.0205 (8)	0.0240 (8)	−0.0004 (6)	0.0064 (6)	0.0011 (7)
C11	0.0260 (9)	0.0217 (9)	0.0267 (9)	0.0038 (7)	0.0076 (7)	−0.0037 (7)
C12	0.0203 (8)	0.0260 (9)	0.0310 (9)	0.0041 (7)	0.0049 (7)	−0.0018 (8)
C13	0.0219 (8)	0.0239 (9)	0.0222 (8)	−0.0018 (7)	0.0050 (6)	0.0019 (7)
C14	0.0269 (9)	0.0206 (8)	0.0251 (9)	0.0006 (7)	0.0082 (7)	−0.0032 (7)
C15	0.0213 (8)	0.0234 (9)	0.0268 (9)	0.0034 (7)	0.0073 (7)	−0.0017 (7)
C16	0.0308 (10)	0.0321 (10)	0.0279 (9)	−0.0026 (8)	0.0015 (7)	−0.0053 (8)
C17	0.0214 (8)	0.0194 (8)	0.0331 (10)	−0.0018 (7)	0.0053 (7)	0.0044 (7)
C18	0.0206 (9)	0.0298 (10)	0.0376 (10)	0.0027 (7)	0.0069 (7)	0.0105 (8)
C19	0.0231 (9)	0.0322 (10)	0.0416 (11)	0.0072 (7)	0.0144 (8)	0.0103 (9)
C20	0.0251 (9)	0.0227 (9)	0.0329 (10)	−0.0024 (7)	0.0118 (7)	0.0042 (8)
C21	0.0212 (9)	0.0250 (9)	0.0334 (10)	0.0014 (7)	0.0044 (7)	0.0054 (8)
C22	0.0192 (8)	0.0230 (9)	0.0368 (10)	0.0027 (7)	0.0068 (7)	0.0012 (8)
C23	0.0340 (10)	0.0352 (11)	0.0326 (10)	−0.0017 (8)	0.0058 (8)	−0.0014 (9)

### Geometric parameters (Å, º)

**Table:**

Cl1—C1	1.8140 (17)	C9—H9C	0.9800
O1—C3	1.212 (2)	C10—C15	1.385 (2)
C1—C17	1.510 (2)	C10—C11	1.401 (2)
C1—C2	1.554 (2)	C11—C12	1.379 (2)
C1—H1	1.0000	C11—H11	0.9500
O2—C7	1.209 (2)	C12—C13	1.392 (2)
C2—C10	1.539 (2)	C12—H12	0.9500
C2—C7	1.549 (2)	C13—C14	1.387 (2)
C2—C3	1.553 (2)	C14—C15	1.394 (2)
O3—C13	1.3669 (19)	C14—H14	0.9500
O3—C16	1.431 (2)	C15—H15	0.9500
C3—C4	1.499 (3)	C16—H16A	0.9800
O4—C20	1.373 (2)	C16—H16B	0.9800
O4—C23	1.429 (2)	C16—H16C	0.9800
C4—C5	1.537 (2)	C17—C22	1.385 (2)
C4—H4A	0.9900	C17—C18	1.404 (2)
C4—H4B	0.9900	C18—C19	1.378 (3)
C5—C8	1.528 (2)	C18—H18	0.9500
C5—C9	1.530 (2)	C19—C20	1.391 (3)
C5—C6	1.536 (2)	C19—H19	0.9500
C6—C7	1.508 (2)	C20—C21	1.383 (2)
C6—H6A	0.9900	C21—C22	1.393 (2)
C6—H6B	0.9900	C21—H21	0.9500
C8—H8A	0.9800	C22—H22	0.9500
C8—H8B	0.9800	C23—H23A	0.9800
C8—H8C	0.9800	C23—H23B	0.9800
C9—H9A	0.9800	C23—H23C	0.9800
C9—H9B	0.9800		
			
C17—C1—C2	117.73 (14)	H9B—C9—H9C	109.5
C17—C1—Cl1	111.52 (12)	C15—C10—C11	118.09 (15)
C2—C1—Cl1	109.52 (11)	C15—C10—C2	121.31 (14)
C17—C1—H1	105.7	C11—C10—C2	120.36 (15)
C2—C1—H1	105.7	C12—C11—C10	120.88 (16)
Cl1—C1—H1	105.7	C12—C11—H11	119.6
C10—C2—C7	108.45 (13)	C10—C11—H11	119.6
C10—C2—C3	106.72 (12)	C11—C12—C13	120.16 (15)
C7—C2—C3	109.30 (13)	C11—C12—H12	119.9
C10—C2—C1	108.18 (13)	C13—C12—H12	119.9
C7—C2—C1	114.51 (13)	O3—C13—C14	124.10 (15)
C3—C2—C1	109.38 (13)	O3—C13—C12	115.89 (15)
C13—O3—C16	117.10 (13)	C14—C13—C12	120.01 (15)
O1—C3—C4	122.46 (16)	C13—C14—C15	119.14 (16)
O1—C3—C2	120.70 (16)	C13—C14—H14	120.4
C4—C3—C2	116.77 (14)	C15—C14—H14	120.4
C20—O4—C23	116.90 (14)	C10—C15—C14	121.72 (15)
C3—C4—C5	112.22 (14)	C10—C15—H15	119.1
C3—C4—H4A	109.2	C14—C15—H15	119.1
C5—C4—H4A	109.2	O3—C16—H16A	109.5
C3—C4—H4B	109.2	O3—C16—H16B	109.5
C5—C4—H4B	109.2	H16A—C16—H16B	109.5
H4A—C4—H4B	107.9	O3—C16—H16C	109.5
C8—C5—C9	109.80 (15)	H16A—C16—H16C	109.5
C8—C5—C6	110.31 (14)	H16B—C16—H16C	109.5
C9—C5—C6	109.66 (14)	C22—C17—C18	117.57 (17)
C8—C5—C4	109.52 (14)	C22—C17—C1	117.99 (15)
C9—C5—C4	109.41 (14)	C18—C17—C1	124.43 (16)
C6—C5—C4	108.10 (14)	C19—C18—C17	120.98 (17)
C7—C6—C5	112.54 (14)	C19—C18—H18	119.5
C7—C6—H6A	109.1	C17—C18—H18	119.5
C5—C6—H6A	109.1	C18—C19—C20	120.32 (16)
C7—C6—H6B	109.1	C18—C19—H19	119.8
C5—C6—H6B	109.1	C20—C19—H19	119.8
H6A—C6—H6B	107.8	O4—C20—C21	124.02 (16)
O2—C7—C6	122.10 (15)	O4—C20—C19	116.11 (15)
O2—C7—C2	121.26 (15)	C21—C20—C19	119.87 (17)
C6—C7—C2	116.65 (14)	C20—C21—C22	119.17 (16)
C5—C8—H8A	109.5	C20—C21—H21	120.4
C5—C8—H8B	109.5	C22—C21—H21	120.4
H8A—C8—H8B	109.5	C17—C22—C21	122.05 (16)
C5—C8—H8C	109.5	C17—C22—H22	119.0
H8A—C8—H8C	109.5	C21—C22—H22	119.0
H8B—C8—H8C	109.5	O4—C23—H23A	109.5
C5—C9—H9A	109.5	O4—C23—H23B	109.5
C5—C9—H9B	109.5	H23A—C23—H23B	109.5
H9A—C9—H9B	109.5	O4—C23—H23C	109.5
C5—C9—H9C	109.5	H23A—C23—H23C	109.5
H9A—C9—H9C	109.5	H23B—C23—H23C	109.5
			
C17—C1—C2—C10	46.58 (18)	C7—C2—C10—C11	−154.85 (15)
Cl1—C1—C2—C10	175.35 (11)	C3—C2—C10—C11	−37.2 (2)
C17—C1—C2—C7	−74.47 (19)	C1—C2—C10—C11	80.41 (18)
Cl1—C1—C2—C7	54.29 (16)	C15—C10—C11—C12	−0.8 (2)
C17—C1—C2—C3	162.47 (14)	C2—C10—C11—C12	−175.36 (16)
Cl1—C1—C2—C3	−68.77 (14)	C10—C11—C12—C13	0.4 (3)
C10—C2—C3—O1	102.75 (17)	C16—O3—C13—C14	5.3 (2)
C7—C2—C3—O1	−140.15 (16)	C16—O3—C13—C12	−174.99 (15)
C1—C2—C3—O1	−14.1 (2)	C11—C12—C13—O3	−179.34 (15)
C10—C2—C3—C4	−74.16 (17)	C11—C12—C13—C14	0.3 (3)
C7—C2—C3—C4	42.94 (18)	O3—C13—C14—C15	179.02 (15)
C1—C2—C3—C4	169.04 (13)	C12—C13—C14—C15	−0.6 (2)
O1—C3—C4—C5	130.01 (17)	C11—C10—C15—C14	0.5 (2)
C2—C3—C4—C5	−53.14 (19)	C2—C10—C15—C14	175.01 (15)
C3—C4—C5—C8	−62.80 (19)	C13—C14—C15—C10	0.2 (3)
C3—C4—C5—C9	176.80 (15)	C2—C1—C17—C22	−92.21 (19)
C3—C4—C5—C6	57.43 (18)	Cl1—C1—C17—C22	139.97 (14)
C8—C5—C6—C7	62.78 (18)	C2—C1—C17—C18	87.3 (2)
C9—C5—C6—C7	−176.16 (14)	Cl1—C1—C17—C18	−40.5 (2)
C4—C5—C6—C7	−56.94 (18)	C22—C17—C18—C19	1.4 (3)
C5—C6—C7—O2	−128.13 (17)	C1—C17—C18—C19	−178.16 (16)
C5—C6—C7—C2	52.09 (19)	C17—C18—C19—C20	0.1 (3)
C10—C2—C7—O2	−106.02 (17)	C23—O4—C20—C21	−3.9 (2)
C3—C2—C7—O2	137.99 (16)	C23—O4—C20—C19	175.34 (16)
C1—C2—C7—O2	14.9 (2)	C18—C19—C20—O4	179.10 (16)
C10—C2—C7—C6	73.77 (18)	C18—C19—C20—C21	−1.6 (3)
C3—C2—C7—C6	−42.22 (19)	O4—C20—C21—C22	−179.16 (16)
C1—C2—C7—C6	−165.33 (14)	C19—C20—C21—C22	1.7 (3)
C7—C2—C10—C15	30.8 (2)	C18—C17—C22—C21	−1.4 (3)
C3—C2—C10—C15	148.44 (15)	C1—C17—C22—C21	178.20 (16)
C1—C2—C10—C15	−93.96 (18)	C20—C21—C22—C17	−0.1 (3)

### Hydrogen-bond geometry (Å, º)

**Table:**

*D*—H···*A*	*D*—H	H···*A*	*D*···*A*	*D*—H···*A*
C8—H8*C*···Cl1^i^	0.98	2.81	3.745 (2)	159
C14—H14···O2^ii^	0.95	2.52	3.394 (2)	153
C19—H19···O3^iii^	0.95	2.56	3.470 (2)	161

Symmetry codes: (i) −*x*−1/2, *y*+1/2, −*z*+1/2; (ii) −*x*, −*y*+2, −*z*+1; (iii) *x*−1, *y*, *z*.
